# Battery-Less NFC Potentiostat for Electrochemical Point-of-Care Sensors Based on COTS Components

**DOI:** 10.3390/s22197213

**Published:** 2022-09-23

**Authors:** Antonio Lazaro, Ramon Villarino, Marc Lazaro, Nicolau Canellas, Beatriz Prieto-Simon, David Girbau

**Affiliations:** 1 Department of Electronics, Electrics and Automatic Control Engineering, Rovira i Virgili University, 43007 Tarragona, Spain; 2 Catalan Institution for Research and Advanced Studies (ICREA), Passeig de Lluís Companys 23, 08010 Barcelona, Spain

**Keywords:** near-field communication, battery-less, potentiostat, electrochemical sensor, point-of-care, glucometer

## Abstract

This work studies the feasibility of using a battery-less Near-Field Communication (NFC) potentiostat for the next generation of electrochemical point-of-care sensors. A design based on an NFC microchip, a microcontroller, and a custom potentiostat based on an operational amplifier is presented. A proof-of-concept prototype has been designed and used to quantify glucose concentration using commercial glucose test strips from chronoamperometry measurements. The device is harvested and the sensor is read using a mobile phone. The prototype uses an antenna loop covered with ferrite sheets to ensure stable operation of the electronics when the mobile phone is used as reader. The use of ferrite reduces the detuning caused by the proximity of the metal parts of the mobile phone. A comparison with a commercial glucometer device is provided. Results obtained using a commercial glucometer and those provided by the proposed potentiostat show an excellent agreement.

## 1. Introduction

In modern societies, there is a progressive aging of the population and an increase in people with chronic diseases that require follow-up care (e.g., diabetes) [1]. In this context, home health care gains increased relevance year after year, underpinned by the growing number of dependent people and mobility restrictions, as well as by the longer life expectancy associated with certain chronic processes. In addition, the Corona Virus Disease (COVID-2019) pandemic has accelerated the need to implement telemedicine services due to the saturation of primary care centers and hospitals [2]. Here, the concept of Point-of-Care Testing (PoCT) arises, defined as the medical diagnostic testing at or near the point of care, in contrast to the historical pattern in which testing was limited entirely or primarily to medical settings [3,4,5]. These testing devices are essential in developing countries to guarantee adequate medical diagnosis in remote areas. The development of this concept in the frame of remote monitoring at home of specific biomarkers by using sensors and communication technologies opens new opportunities and a new paradigm in the digital transition in the field of health. To this end, low-cost wearable devices pave the way for the new generation of PoCT devices [6]. These devices should be easy to use for seniors.

Electrochemical biosensors, especially those based on electrochemical transduction, can meet most of those requirements. Electrochemical biosensors are characterized by the high sensitivity and selectivity that they can achieve by using a small sample volume. The electrochemical transduction relies on a potentiostat, which is an electronic instrument capable of measuring the current flowing through a chemical cell, which depends on the voltage applied between its electrodes. In order to replace commercial expensive potentiostats, low-cost and open-source potentiostats have been recently presented in the literature [7,8,9,10]. However, to control, process, and monitor the results of these instruments, a computer is necessary. These portable devices are preferably intended to be used by scientists replacing expensive laboratory instruments rather than as PoCT devices, where the computer and screen can be replaced by a smartphone. Recently, potentiostats connected to a smartphone via Bluetooth [11], or Wi-Fi. Additionally, USB or audio port interfaces [11] have been used to power the device from the smartphone. However, these devices require either cables or batteries to power the wireless transceiver, increasing the cost of the final device and the difficulty to integrate them into cheap wearable devices. In addition to clinical diagnosis, electrochemical sensing has been widely applied to other fields such as food safety or environmental monitoring [12].

From a connectivity point of view, Radio Identification (RFID) is one of the most important key enabling technologies for massive deployment of Internet of Things (IoT). RFID is a system made up of tags whose electromagnetic response can be received by specialized readers. Although the technology has been available for more than two decades, it has not been oriented to the retail sector until recent years. A RFID subset such as Near Field Communication (NFC) has recently been massively incorporated for payment (especially due to the COVID pandemic) and consequently, most smartphones incorporate NFC readers. In addition, the potential of this technology is growing in other fields such as in the development of wireless sensors. NFC sensors that can be read from smartphones have great potential in the IoT thanks to the ease of accessing the data cloud, identifying an object, obtaining its location or sensing some parameters. Such approach has an enormous potential in a wide range of applications [13,14].

While the use of Bluetooth is a moderate/high-cost solution, it needs batteries and allows a working range of up to a few meters, body-worn wearable sensors based on passive UHF-RFID technology is limited to distances of approximately one meter [15] due to the losses on the antenna caused by the proximity of the body. In addition, UHF RFID Integrated Circuits (IC) with sensing capabilities have higher sensitivity than that of integrated circuits designed for inlay applications [14]. Passive UHF-RFID tags do not need batteries, but the main drawback is that the smartphone cannot work as a reader unit. The use of smartphones as readers creates a fast and ubiquitous interface with the data cloud that enables simple and low-cost applications within the context of IoT. On the other hand, ad hoc readers are too expensive for a number of applications that are expected to be widely used in the future. NFC technology can solve these problems. NFC-based battery-free electrochemical sensors can harvest radio frequency energy to acquire and transmit data to a smartphone over a short distance, making them suitable as an enabling technology for PoCT. As a result, various battery-less NFC-based sensors have been proposed in the literature for several applications [16,17] including biomedical ones [14,18,19].

Potentiometric [6,20] as well as colorimetric-based NFC sensors have already been proposed [21,22]. Advanced sensors based on smartphone have been recently reported [23,24,25]. Recently, a NFC Integrated Circuit (IC) including an integrated potentiostat has been proposed in [26,27], highlighting the interest in this technology.

In this work, the feasibility of designing a pontentiostat using NFC technology is demonstrated. The design consisting of commercial components enables fast chronoamperometric measurements. The use of a custom pontentiostat allows to extend the bias ranges of the circuits compared to other integrated IC implementations [26]. The integrated microcontroller allows for the implementation of advanced bias sequencies required for some sensors such as commercial glucose test strips. A ferrite-based antenna has been designed to achieve stable power supply of the sensor components, thus being able to perform the measurements correctly, in addition to achieving greater robustness against the effects of detuning caused by the proximity of the metal parts of the mobile.

The paper is organized as follows. Section 2 introduces electrochemical sensors and the fundamentals of chronoamperometry. Section 3 describes the proposed NFC system focusing on the antenna coil and the pontentiostat designs enabling current measurements. A glucose sensor based on commercial test strips is implemented with the designed NFC pontentiostat as a proof of concept of low-cost PoCT NFC-based system and it is presented in Section 4. Experimental results are shown in Section 5. Finally, conclusions are provided in Section 6.

## 2. Electrochemical Sensors

Electrochemical sensors are usually based on a two- or three-electrode electrochemical cell. For amperometric measurements, added to the working or active electrode, the electrodic system also includes a reference electrode and a counter electrode. Most electrochemical-based PoCT devices rely on amperometric measurements to quantify the concentration of a target analyte in a sample. Amperometric sensors provide an electrical current which is proportional to the concentration of the analyte. Amperometry can be easily implemented using a low-cost meter and the analysis of the obtained data is simpler and faster than other electrochemical techniques such as Electrochemical Impedance Spectroscopy (EIS). Amperometric techniques consist of measuring the current flowing through the cell by exciting it with a voltage step at its input. The applied voltage is often determined from voltammetry sweep.

**Figure 1:**
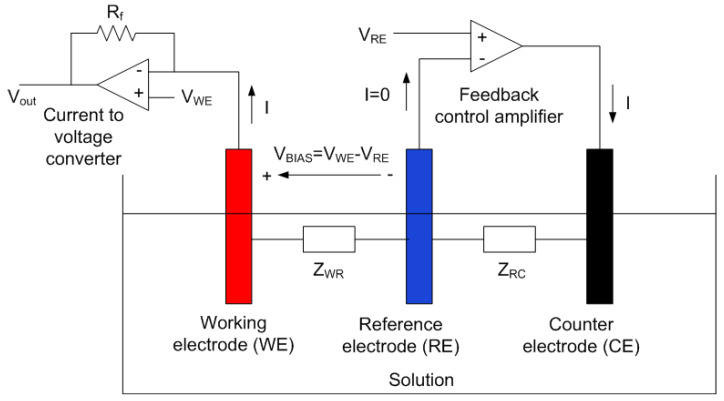
Schematic of a potentiostat, with the electrochemical cell modeled by two equivalent circuits with their respective impedances.

The current measurements in sensors based on three-electrodes are carried out by means of a potentiostat. Figure 1 shows a simplified schema. The cell consists of a working (WE), counter (CE) and reference (RE) electrodes. When an electric potential is applied between the WE and CE, a redox reaction takes place in the electrolyte and the resulting current is measured on the working electrode. The potentiostat is an electronic circuit whose function is to maintain the potential of the working electrode at a constant value with respect to the reference electrode by adjusting the voltage at the counter or auxiliary electrode using a control amplifier while measuring the current flow.

**Figure 2:**
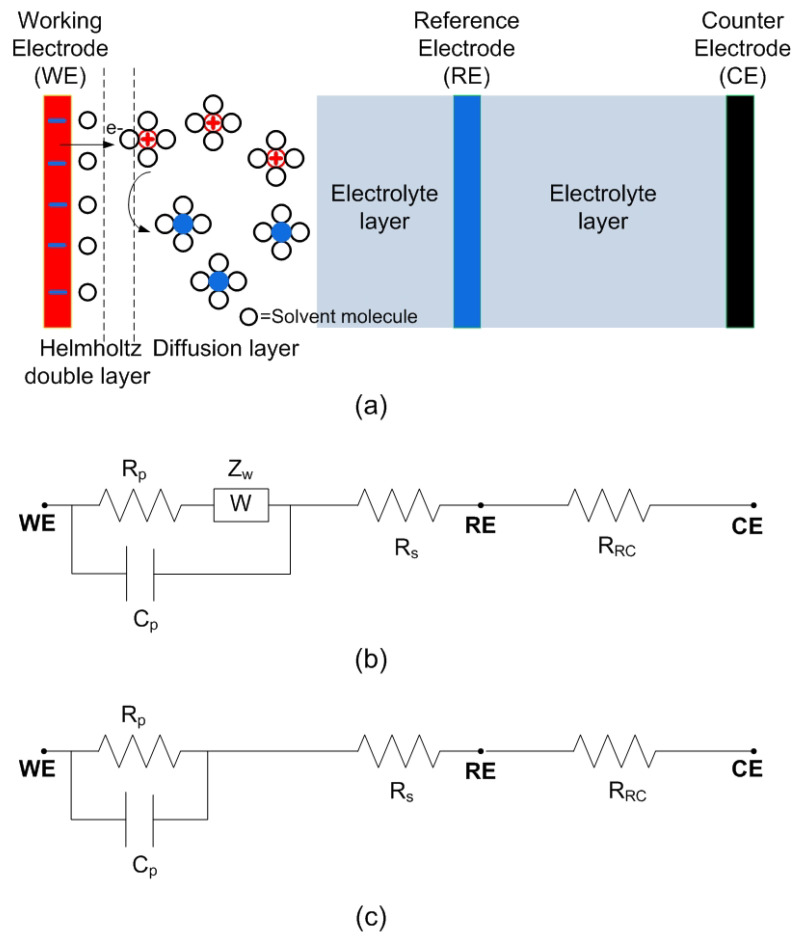
Modeling of the electrochemical cell. (**a**) Diagram of the processes at the electrode-electrolyte interface.The oxidants (red) with a positive charge diffuse toward the negatively charged electrode, accept electrons from the electrode at the interface, become the reductants (blue), and diffuse to the bulk of the solution. The dashed lines are the inner and outer Helmholtz planes, respectively. Equivalent circuit model (Randles equivalent circuit): (**b**) including the Warburg element that takes into account the controlled diffusion process in the low frequency region, and (**c**) without the Warburg element.

In order to understand the potentiostat operation and to verify its operation a simplified equivalent circuit of the electrochemical cell [28,29] is considered in Figure 2. The equivalent electrical circuit of the RE-CE branch ($Z_{RC}$) is a resistor ($R_{RC}$) that models the resistance of the electrolyte between the reference and counter electrodes. A typical Randles equivalent model [30,31] for the electrode-electrolyte interface and the relation between the components and the processes are schematically described in Figure 2. In the model of Figure 2, $R_{s}$ is a parasitic resistance associated with the electrolyte layer between the diffusion layer and the reference electrode. An electrical double layer exists on the interface between the working electrode and its surrounding electrolyte. This double layer is formed when ions from the solution are adsorbed onto the electrode surface. The charged electrode is separated from the charged ions by an insulating space, often on the order of angstroms [29]. These charges separated by an insulator form a capacitor. The interface between the working electrode and the electrolyte is modeled by the double-layer capacitance $C_{p}$, in parallel with the faradaic impedance $Z_{F}$. The faradaic impedance has two terms: $R_{p}$ is the polarization resistance or charge-transfer resistance, which is series-connected with the Warburg impedance $Z_{w}$. Warburg impedance models the diffusion (that arises from the mass transfer) process. $Z_{w}$ is given by [31]:

(1) $$Z_{w}=\frac{(1-j)\sigma }{\omega^{1/2}}$$

where σ is the Warburg diffusion coefficient of the ions in the electrolyte. At high frequencies the Warburg impedance can be neglected ($Z_{w}\to 0$ when ω→∞), resulting in the simplified Randles model of Figure 2c. Usually, the equivalent circuit of an electrochemical cell is more complicated and is determined by adjusting the impedance measured with an electrochemical impedance spectroscopy (EIS) measurement. However, the simplified Randles equivalent circuit is a good starting point for understanding the cell operation and to design dummy cell for testing the potentiostat.

**Figure 3:**
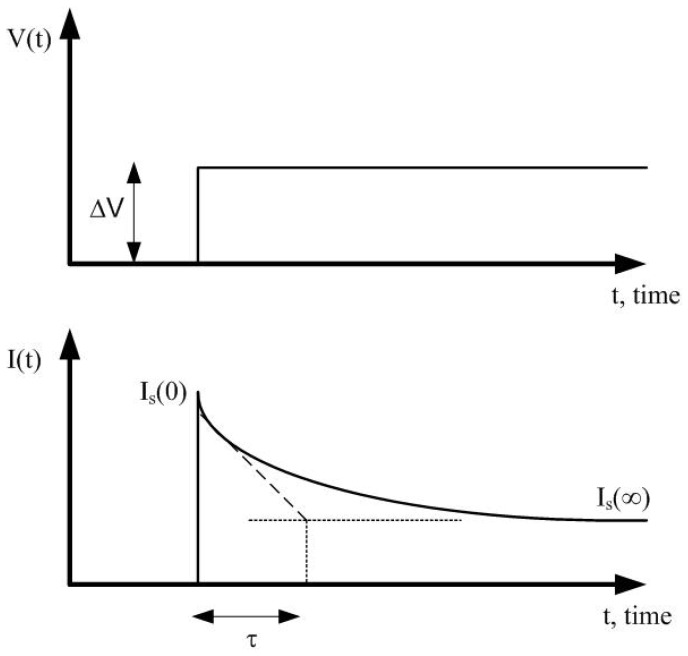
Current response to a voltage step applied at the input bias between the working and reference electrodes.

Assuming the Randles equivalent circuit (Figure 2c), the current response, $I_{s}\left(t\right)$, to a voltage step excitation between the working and reference electrodes, ΔV can be analyzed (Figure 3):

(2) $$I_{s}\left(t\right)=I_{s}\left(0\right)+\left(I_{s}\left(\infty \right)-I_{s}\left(0\right)\right)\left(1-exp(-t/\tau )\right)$$

where $I_{s}\left(0\right)$ is the initial current at t=0, which depends on the electrolyte resistance and is independent from the concentration because at the initial instant the capacitor is short-circuited:

(3) $$I_{s}\left(0\right)=\frac{\Delta V}{R_{s}}$$

The steady-state value of the current when t→∞ (final value when the capacitor is an open circuit) is given by:

(4) $$I_{s}\left(\infty \right)=\frac{\Delta V}{R_{s}+R_{p}}$$

The time constant is:

(5) $$\tau =C_{p}\frac{R_{s}R_{p}}{R_{s}+R_{p}}$$

Note that the final value and the time-constant depend on the charge-transfer resistance $R_{p}$ and therefore on the concentration. In amperometry, the measurement of the final value of the current (when it is considered that current is stabilized) is used to determine the analyte concentration after a calibration procedure where an equation of a dose–response curve is often used. The dose–response curve is obtained experimentally by measuring the final value of the current for solutions with different concentrations. Due to nonlinear behavior of the electrochemical sensors, the equivalent circuit components are a function of the bias voltage and the step voltage. In this work, the Randles equivalent circuit is used to develop a dummy cell for testing the potentiostat by comparing the theoretical current (2) to an input step voltage with a known equivalent circuit. The current range is controlled with the resistance $R_{s}$ and $R_{p}$, and the time response with the capacitance $C_{p}$. Results will be presented in Section 5.

## 3. System Overview and Theoretical Background

### 3.1. System Overview

**Figure 4:**
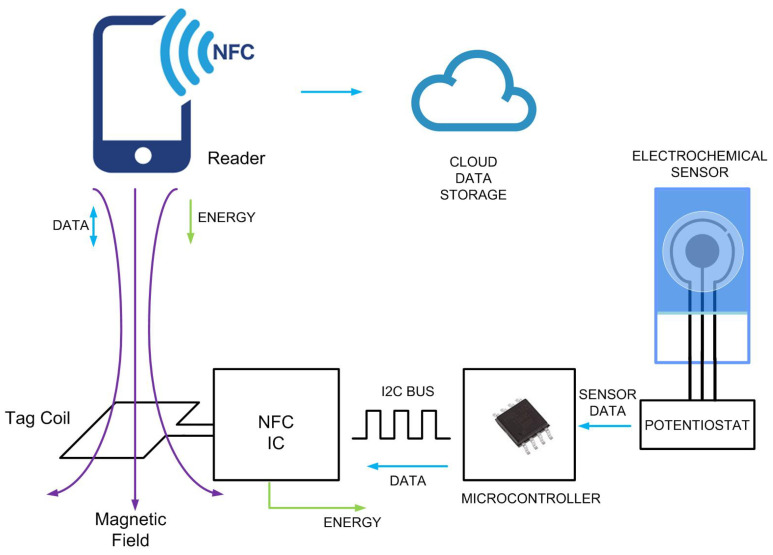
Scheme of the system.

The proposed system is composed of an NFC IC with energy harvesting capability (ST25DV04K from STMicroelectronics), the NFC antenna, a microcontroller (ATTiny1614, from Microchip), a custom pontentiostat, and the electrochemical sensor. A scheme of the system is shown in Figure 4. The ST25DV04K complies with the ISO/IEC 15693 standard or NFC forum type 5, which defines the modulation parameters, and has energy harvesting capability. Authors have already demonstrated that this device allows to harvest more than unregulated 3 V and 3 mA, using a square-shaped printed loop with dimensions of up to 3 cm on each side [32]. The NFC IC has two purposes: to collect the energy coming from the reader to power up the sensor and to establish data communication between the sensor and the reader. Communication between the reader and the tag is based on electromagnetic induction between two loop antennas and works in the 13.56 MHz ISM band. Therefore, external circuits can be powered using the energy harvesting output without the need for a battery. The low-power microcontroller ATTiny1614 is used to read the voltage output of the readout sensor circuit (pontentiostat), by means of its internal 10-bit analogue-to-digital converter (ADC). The ATtiny1614 is an 8-bit AVR microcontroller with 16 kB Flash memory that incorporates an 8 bit digital-to-analogue converter (DAC). This memory is enough to develop custom firmware required for PoCT applications. Current consumption is about 600 μA at 1 MHz, 2.5 V, assuming that the ADC is active. The bias voltage applied on the electrochemical sensor is configured from the DAC. The data read by the microcontroller is sent to the EEPROM memory of the NFC IC through a two-wire $I^{2}C$ serial interface. The data is encoded in a standard NFC data exchange format (NDEF) that is readable using NFC protocol. A mobile phone with NFC capability (e.g., Xiaomi Readmi Note 9 Pro) is used to read the NDEF message stored in the NFC IC memory, perform the calculations and upload the data to the cloud. The PCB prototype that includes the electronics is manufactured on a 0.8 mm thick FR4 substrate, with relative permittivity of 4.7 and loss tangent (tanδ) of 0.02. The cooper metallization layer is 35 μm thick (conductivity $\sigma =4.7\times 10^{6}$ S/m).

The sensor tag is read with an NFC smartphone. Therefore, the energy harvesting should be optimized for low intensity electromagnetic fields. Under these conditions and depending on the chosen NFC antenna, the NFC IC can deliver an output power of 20 mW [32]. The energy is harvested from the reader, therefore the read range of these tags is reduced compared to those without energy harvesting. To optimize energy harvesting, two main guidelines shall be considered: minimize the load current needed to be harvested and improve the power conversion efficiency between the reader and the tag. The element of the tag with the highest energy consumption is the microcontroller. To reduce the total consumption, a microcontroller frequency of 1 MHz and a supply voltage of 2.5 V are chosen, enough for the CMOS amplifiers used (OpAmp) to work, allowing to achieve an adequate dynamic range to polarize the sensors. The OpAmp current consumption depends on the required gain bandwidth product. Low-current range measurements require high gains and therefore OpAmp with better performance in terms of gain are needed. Calculations are performed using a smartphone that is also used to display the results. Therefore, the tag is mainly responsible for performing the measurements and only basic operations. In addition, this fact allows to reduce the number of components, size and cost of the tag.

The NFC energy harvesting output ($V_{EH}$) also powers the $I^{2}C$ bus, which requires pull-up resistors on both the clock (SCL) and data (SDA) lines. These pull-up resistors must be designed to limit the sink current when any of the lines are pulled down. To reduce current consumption 20 kΩ are used in the prototype.

The reader (smartphone) starts interrogating the tag cyclically using different protocols waiting for the response of the tag, because its standard is not known a priori. Therefore, the communication between reader and tag employs ASK modulation.The reader determines which modulation index is used and the commands must be decoded independently from the value used by the tag. The value of the capacitor $C_{EH}$ connected to the energy harvesting NFC output must be chosen to avoid voltage drops during the modulation pauses of the reader (pulse modulation type) whose duration is $T_{p}$. The worst case occurs when the mobile is in the interrogation state when searching for tags and a 100% ASK modulation is used, because the reader does not transmit the carrier during the pause period, and therefore the rectified voltage and the energy harvesting output decrease. Commercially-available NFC ICs with energy harvesting are based on ISO14443-A and mainly on ISO 15693. Other standards such as ISO/IEC 18092 (e.g., FeliCa) recognized by NFC forum are mainly used in RFID contactless payment systems (which is very popular in Japan). In both standards, ISO 14443-3 and ISO 15693, $T_{p}$ is approximately 10 μs. Therefore, a low-drop regulator reduces the voltage fluctuations in reader interrogation periods. The voltage drop can be estimated from the discharge in the storage capacitor:

(6) $$V_{drop}=\frac{I_{L}\cdot T_{p}}{C_{EH}}$$

where $I_{L}$ is the load current obtained from the energy harvesting output. When a value of $C_{EH}=1\;\mu$F is chosen, a voltage drop of 20 mV is generated at the regulator input, which is acceptable for low-drop regulators as long as the load currents are less than 2 mA. Note that this dropout is like a small burst that can propagate to the Analogue Front-End (AFE) introducing noise that is amplified in the potentiostat’s high-gain Transimpedance Amplifier (TIA). To filter the signal at the output, a capacitor is added between the inverting input and the output of the TIA amplifier. In this work, the LMS5214IMG-2.5 regulator from Texas Instruments was used. This regulator has a maximum current consumption of 10 μA and has a voltage dropout of 0.2 V. In general, the voltage output of the NFC IC used provides an unregulated 3 V that is compatible with the voltage dropout of the regulator, which leaves a certain safety range in case of misalignment between the coils of the reader and the tag or in case of detune effects.

### 3.2. Antenna Design and Tag Tuning

**Figure 5:**
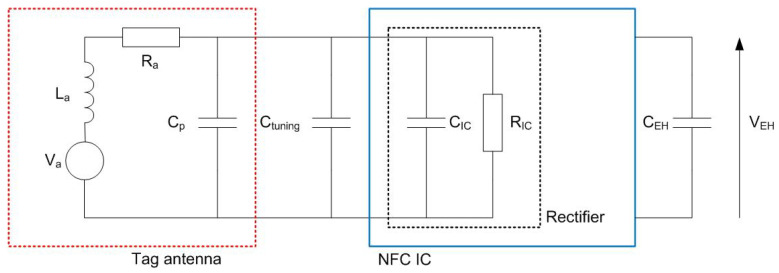
Equivalent circuit of the tag antenna and NFC IC.

To achieve high energy transfer, the resonant frequency of the tag must be adjusted to the operating frequency of the NFC (13.56 MHz). To this end the simplified equivalent circuit of Figure 5 can be used. In this equivalent circuit the impedance of IC chip is modeled as a parallel RC circuit, where $R_{IC}$ is the nonlinear IC resistance and $C_{IC}$ is the chip capacitance. The equivalent electric circuit of the antenna in the tag consists of an inductance $L_{a}$ with a series resistance $R_{a}$ that models the antenna losses (DC plus AC due to skin effect). The parallel capacitance $C_{p}$ is associated with parasitic capacitance of antenna and layout interconnections. The tag’s resonance frequency is given by:

(7) $$f_{r}=\frac{1}{2\pi }\sqrt{\frac{R_{IC}+R_{a}}{L_{a}R_{IC}\left(C_{IC}+C_{p}+C_{tuning}\right)}}\approx \frac{1}{2\pi \sqrt{La(C_{IC}+C_{p}+C_{tuning})}}$$

where $C_{tuning}$ is an external capacitor connected between the antenna pins of the NFC IC, whose function is to adjust the resonance frequency. The chip capacitance is nonlinear but it is nearly constant for typical input power ranges. For the ST25DV04 its value is 28.5 pF. The parasitic $C_{p}$ is of the order of few pF. $R_{IC}$ is high nonlinear and it is of the order of several hundred Ω to kΩ [33,34].

In order to increase the read range or the load current that the energy harvesting output can supply, the average magnetic field in the tag antenna should be higher than the minimum value known as $H_{min}$. This value corresponds to the minimum voltage required to wake-up the tag for the desired load conditions ensuring the correct working of the RF rectifier. The average field can be obtained from the Faraday law, the AC variation of the magnetic flux induces a voltage at the antenna coil terminals, and analyzing the circuit of Figure 5 [14]:

(8) $$H_{min}\approx \frac{\sqrt{{\left(1-{\left(f/f_{r}\right)}^{2}\right)}^{2}+1/Q_{T}^{2}}}{2\pi f\mu_{ef}A\cdot N}\cdot V_{min}$$

where *A* is the loop antenna area, *N* is the number of loops, $\mu_{ef}$ is the effective magnetic permeability (equal to the vacuum $\mu_{0}$ for antennas in the air without ferrites), $V_{min}$ is the minimum voltage required for the NFC IC and $Q_{T}$ is the total quality factor of the tag at the resonance frequency given by:

(9) $$Q_{T}=\frac{1}{\frac{R_{a}}{2\pi f_{r}L_{a}}+\frac{2\pi f_{r}L_{a}}{R_{IC}}}\approx \frac{R_{IC}}{2\pi f_{r}L_{a}}$$

which is the combination of the quality factors of the antenna coil and of the IC. By analyzing (8), some guidelines to optimize the tag can be considered. Larger antenna size allows to reduce the minimum magnetic field. It is essential to reduce the detuning of the tag caused by the presence of metal parts such as the mobile phone case, which reduces the inductance of the tag due to the image currents induced in the metal [14,35]. Increasing the number of turns can be counterproductive, since the losses of the tag antenna increase and, consequently, the quality factor worsens. However, the total quality factor is mainly determined by the input resistance of the IC.

When the number of turns is increased, the antenna inductance increases as $N^{2}$ (see for example the Wheeler’s formula for inductance [36]):

(10) $$L=K_{1}\mu_{0}N^{2}\frac{D_{av}}{1+K_{2}\rho }$$

where $\mu_{0}$ is the vacuum magnetic permeability constant ($4\pi \cdot 10^{-7}$ H/m) and $D_{av}$ is the average diameter between the outer diameter and inner diameter:

(11) $$D_{av}=\left(D_{out}+D_{in}\right)/2$$

and ρ is the fill factor represents how hollow the inductor is, and it is defined as:

(12) $$\rho =\frac{D_{out}-Din}{D_{out}+D_{in}}$$

The coefficients $K_{1}$ and $K_{2}$ are shape dependent and for a square-shaped inductor are 2.34 and 2.75, respectively.

Losses in a coil are fundamentally caused by the series resistance, which at high-frequency increases due to the skin depth [37]. The antenna resistance increases approximately proportional to the length and therefore with the number of turns. In consequence, the quality factor of the antenna increases with N. However, the antenna quality factor for typically printed antennas (using conductors with a thickness higher than the skin depth) is higher than the load quality factor, which basically depends on $R_{IC}$. Thus, in this case, the total quality factor $Q_{T}$ (9) is inversely proportional to the inductance $L_{a}$ and is proportional to $N^{2}$. If the tag is tuned at the resonance frequency, the value of $H_{min}$ will be proportional to the antenna inductance $L_{a}$ divided by *N*, and therefore proportional to the number of turns. A minimum value of inductance must be considered because the total quality factor is limited by the bandwidth (two times the subcarrier frequency used in the communication between the tag and the reader) [14]. The antenna together with the parasitic elements and the input impedance of the chip works as a bandpass filter. If the total quality factor of the tag was too high, the sidebands would be attenuated and the communication between tag and reader would be degraded. The maximum value of the quality factor can be estimated from the bandwidth using the following relationship:

(13) $$Q_{Tmax}=\frac{f_{0}}{BW}\approx \frac{f_{0}}{2f_{sideband}}$$

where $f_{0}$ = 13.56 MHz is the tag frequency operation, and $f_{sideband}$ is the frequency of the modulating subcarrier, that is a function of the standard. For tags under ISO 14443 ($f_{sideband}=f_{0}/16$) and ISO 15963 ($f_{sideband}=f_{0}/28$), the maximum total quality factor is 8 and 14, respectively.

To understand the influence of the antenna size and the number of turns, simulations using the analytical inductance expression (from modified Wheeler’s formula) for a square-shaped antenna [36], with strips width *w* and separation between them *s* 0.5 mm, have been performed. In these simulations, the antenna quality factor can be evaluated from the equivalent series resistance that can be computed from the wire resistance taking into account the skin depth [37]:

(14) $$R_{a}=\frac{L_{c}}{\sigma \cdot w\cdot \delta (1-exp(-t/\delta ))}$$

where *t* is the conductor thickness (34 μm in the simulations), σ the conductor conductance ($4.7\times 10^{7}$ S/m for Cooper) and $L_{c}$ is the total length of the conductor. For a square-shaped loop antenna the total length of conductor is

(15) $$L_{c}=4ND_{out}-4Nw-{\left(2N+1\right)}^{2}\left(s+w\right)$$

The skin depth is given by [37]:

(16) $$\delta =\frac{1}{\sqrt{\sigma \mu \pi f}}$$

**Figure 6:**
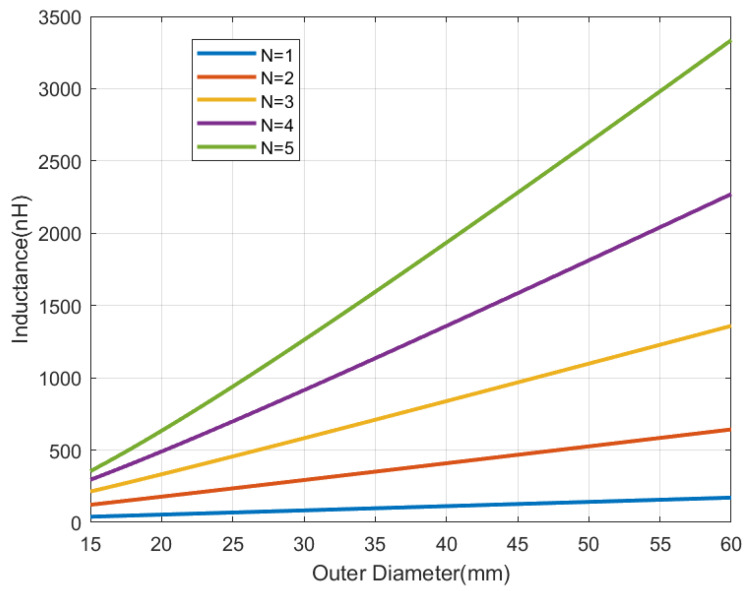
Simulated inductance as a function of the outer diameter for a square antenna with a different number of turns.

**Figure 7:**
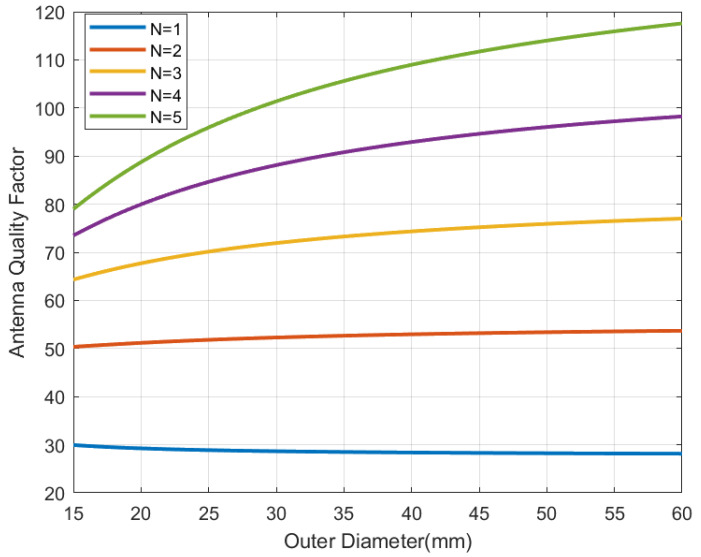
Simulated antenna quality factor as a function of the outer diameter for a square antenna with a different number of turns.

**Figure 8:**
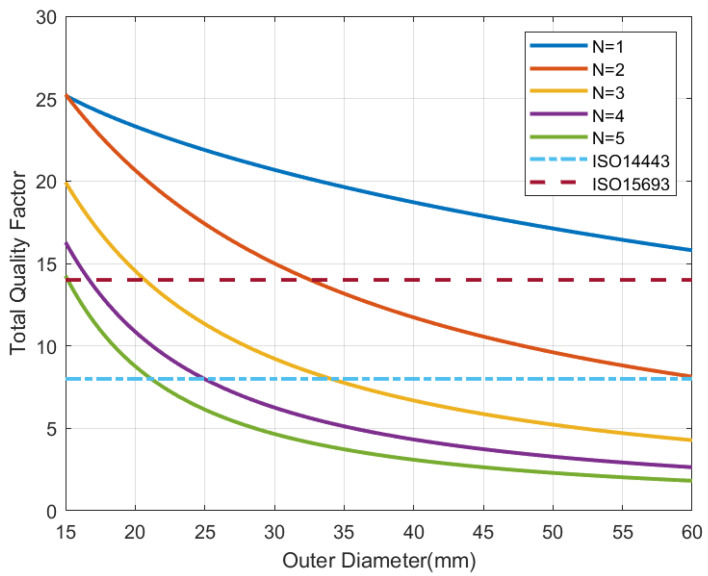
Simulated total tag quality factor as a function of the outer diameter for a square antenna with a different number of turns.

**Figure 9:**
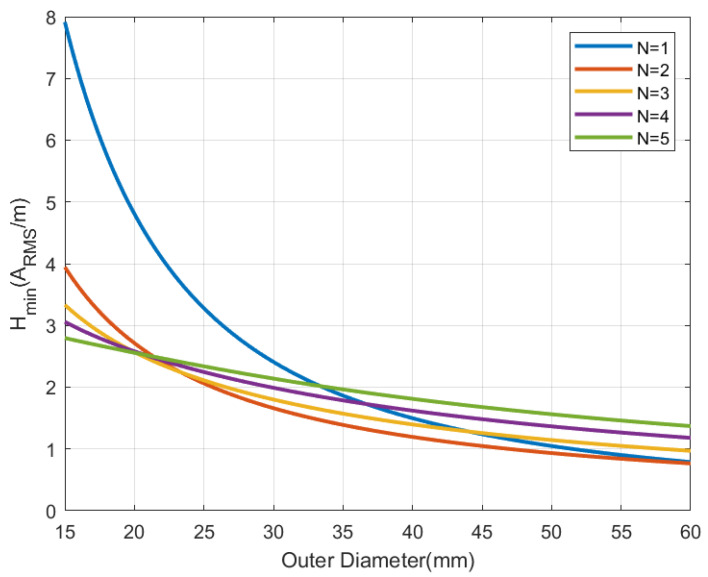
Simulated minimum magnetic field as a function of the outer diameter for a square antenna with a different number of turns.

**Figure 10:**
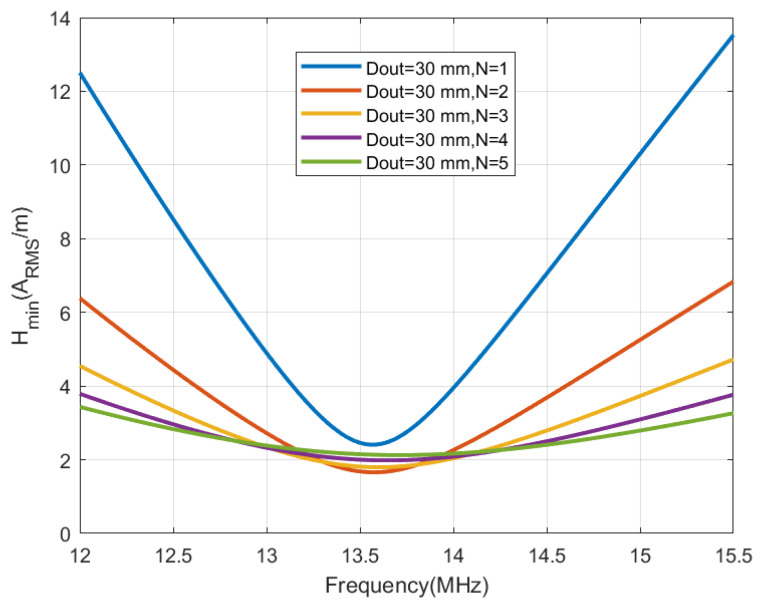
Simulated minimum magnetic field as a function of the frequency for a square antenna with outer diameter of 30 mm and a different number of turns.

Figure 6, Figure 7, Figure 8 and Figure 9 show the inductance, the antenna quality factor ($Q_{a}$) and the total quality factor ($Q_{T}$) computed from (9) and the minimum magnetic field (8) considering $R_{IC}$ = 525 Ω, as a function of the outer diameter of the coil for a different number of turns. Figure 8 includes the maximum total quality factor based on the standard ISO 14443 or ISO 15693. Figure 9 shows that increasing the number of turns can penalize the reading range of the tag and the energy harvesting for coils with diameters higher than 22 mm, considering the simulated conditions. The total quality factor also controls the detuning sensitivity (8). Figure 10 shows the $H_{min}$ as a function of the frequency for a 30 mm outer diameter coil ($D_{out})$ and different numbers of turns. The minimum magnetic field should be higher when the tag is detuned due to the tolerance of component values or the presence of metal nearby. The resonance frequency will be different from the operating frequency (13.56 MHz). Finally, a small inductance value generates higher currents that circulate through the tag, and therefore, can produce loading effects on the reader, worsening its impedance matching for short distances where the coupling coefficient between reader and tag is larger [38]. Therefore, if the number of turns is increased, although the value of $H_{min}$ is penalized a little, the influence of the non-linear IC resistance variations is reduced in case of modifying the reader-tag distance.

To increase the energy transfer, the coupling coefficient between the tag and reader antenna should be as high as possible. To this end, the size of the tag antenna should be close to that of the reader antenna, but not the same size to avoid detuning effect at short distances [39]. Modern mobiles integrate the NFC antenna around the camera aperture or over the battery case with typical sizes being about 2–2.5 cm${}^{2}$ [40,41,42,43].

Taking into account the last considerations, the quality factor is limited by the IC load. From (8) the minimum inductance for ISO 15693 is about 440 nH considering a typical $R_{IC}$ of 525 Ω and assuming the NFC IC has the energy harvesting activated, which will allow it to power the electronics of the tag [32]. However, this small inductance value generates high currents through the tag that loads the transmitter causing mismatches when the tag is close to the mobile and therefore the coupling coefficient is high as expected in this application. Therefore, a higher inductance avoids this drawback and gives more robustness to the system under nonlinear variations of the NFC IC resistance. In this work, a square loop of side 3 cm is used as the antenna for the prototype. The number of loop turns is five, with a width of the printed conductors and a separation between them of 0.5 mm in both cases. The substrate used is FR4 (thickness 0.5 mm, dielectric constant 4.7, dissipation losses of 0.02 and copper metallization). In fact, the substrate has a relative influence, thus, if the application requires it, other substrates including flexible ones could be used. Since the substrate is a non-magnetic material, its permittivity affects the value of the parasitic capacitance of the antenna. The thickness of the metallization and the width and length of the printed conductors determine the antenna resistance. The final size is similar to that of an epidermal patch or that of a smartwatch, and it is close to that of a mobile NFC antenna. In addition, it includes the electronics for the sensor. Due to the presence of metal parts such as the PCB ground plane or the own electronic part, different configurations are considered. The antenna can be printed together the electronic circuits on the same substrate, although this means a increase in the size of the tag. To reduce the size of the tag, the antenna must be separated from the electronics. Various configurations can be considered. The first one consists of an antenna slightly separated from the ground plane of the PCB to avoid the undesired effect of the metal on the inductance. The second configuration adds sintered ferrite foil (MULL12060-200 model from Laird Technologies) below the antenna to improve tag performance in the presence of the smartphone and a ground plane. The flexible ferrite sheet is 0.1 mm thick with complex relative magnetic permeability of 150-j5 at 13.56 MHz.

A loop antenna prototype has been manufactured and connected to SubMiniature version A (SMA) connector to be characterized with the Vector Network Analyzer (VNA). Once the reflection coefficient has been measured, the impedance is obtained, from whose imaginary part the inductance is determined. The quality factor is computed from the ratio between the imaginary and the real part of the measured impedance.

**Figure 11:**
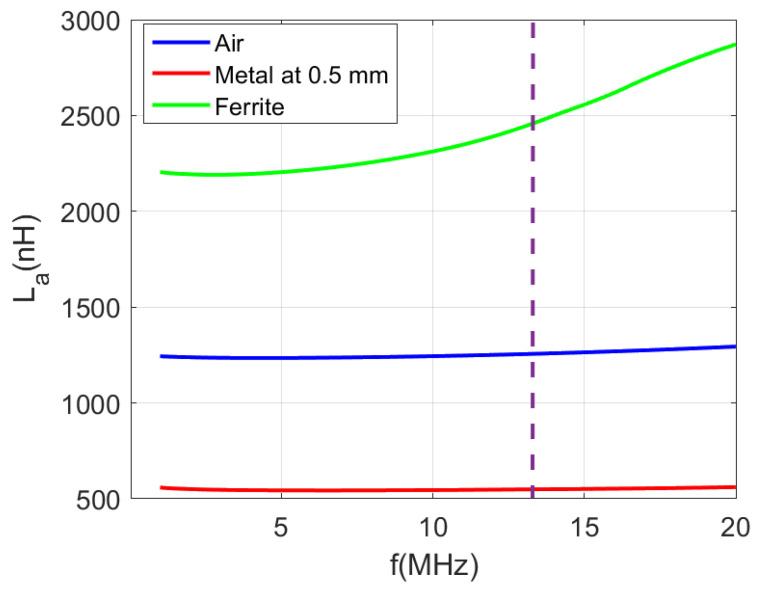
Measurement of the inductance of a loop antenna for different cases: in the air, separated from a metal by 0.5 mm and covered with a ferrite foil on a ground plane.

**Figure 12:**
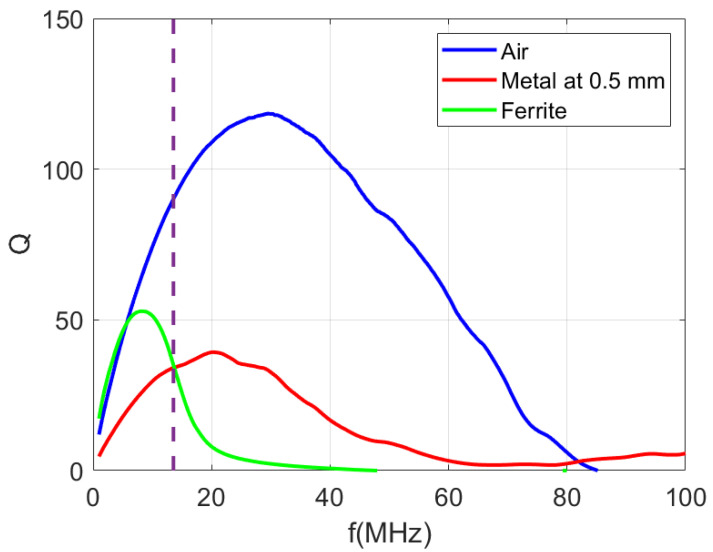
Measurement of the quality factor of a loop antenna for different cases: in the air, separated from a metal by 0.5 mm and covered with a ferrite foil on a ground plane.

Figure 11 and Figure 12 show the measured inductance and quality factor as a function of the frequency. Table 1 summarizes some parameters for the three cases that are experimentally studied: the antenna in the air, the antenna close to the ground plane of the PCB and the antenna near the PCB itself covered with sintered ferrite foil on both sides. A significant reduction in inductance can be observed when the metallic plane is closer. Therefore, the proximity of the metal case of the mobile phone can detune the antenna. On the other hand, the antenna including ferrite has a higher inductance than in air (approximately double). The quality factor values for frequencies below 10 MHz are similar to those achieved by the antenna in free space. However, the quality factor decreases as the frequency increases. Finally, a quality factor of about 35 is obtained in the presence of metal, provided that the prototype is coated with ferrite. Although this value may seem low, it must be remembered that the total quality factor is determined by the external quality factor of the IC, leading to similar values for the three configurations (see (9)). This consideration is important, so it is possible to use antennas with low quality factors since the IC mainly determines the total quality factor. However, the antenna with ferrite is more robust against detuning caused by the proximity of the cell phone. This point is crucial because the mobile phone will be over the tag for a few seconds during the measurement, and the power supply must be enough to keep the electronics of the tag active during the measurement. To avoid the detuning of the antenna, the distance from the mobile must be of the order of one cm, reducing the wireless power efficiency due to the small value of the coupling coefficient between the antennas, which decreases with the distance. In addition, the variation of the distance during the measurement can produce an undesired drop of voltage that can affect the sensor measurement. Therefore, in this application, the mobile is expected to be on top of the tag for the duration of the measurement. The use of a ferrite sheet prevents the effect of the detuning. The resonance frequency decreases when the inductance increases but remains above the operating frequency (13.56 MHz). For the different configurations, a surface mount device (SMD) tuning capacitor can be used to adjust the resonance frequency of the tag according to (7). In order to take into account all the parasitic capacitances, the value can be fine adjusted with a test loop connected to the VNA. To do this, the reflection coefficient is measured by checking the value of the resonance frequency.

### 3.3. Potentiostat

**Figure 13:**
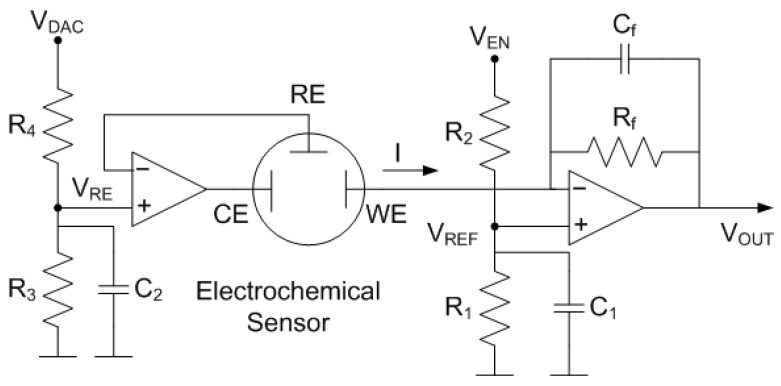
Electrical scheme of the potentiostat.

The Analog Front-End (AFE) that interfaces directly with the electrochemical cell is a potentiostat [7,8,9,10]. In order to reduce the power consumption of the potentiostat, a circuit based on two single-biased Operational Amplifiers (OpAmp) is used (see Figure 13). The reference electrode is set to a voltage $V_{RE}$ obtained from a resistive divider connected to one of the microcontroller’s Digital-to-Analog Converter (DAC) outputs ($V_{DAC}$). Therefore, it can be adjusted. In turn, the working electrode is set to a $V_{REF}$ that is obtained from a resistive voltage divider connected to a digital output of the microcontroller $\left(V_{EN}\right)$. The second OpAmp is configured as a Transimpedance Amplifier (TIA). The objectives are to amplify the current flowing between CE and WE through the feedback resistance $R_{f}$ and to convert it to an output voltage $V_{out}$. This design based on OpAmps takes advantage of some its characteristics such as the negligible input current, the high input impedance and the low input offset voltage. In this work, the low-noise Rail-to-Rail CMOS dual OpAmp TI OP2314 has been chosen. It is characterized for a 3 MHz Gain Bandwidth product, slew-rate of 1.5 V/us, 130 μA per channel and can work from 1.8 V. The DAC output ($V_{DAC}$), and the digital output ($V_{EN}$) are triggered by the microcontroller at same time to generate a voltage step. Bypass capacitors $C_{1}$ and $C_{2}$ have been included to filter noise from the supply. $C_{f}$ is a feedback capacitor whose function is to low-pass filter the signal to reduce noise at the output. The cut-off frequency of the filter is calculated using the following expression:

(17) $$f_{c}=\frac{1}{2\pi R_{f}\cdot C_{f}}$$

Its value is selected to let the frequency components of interest to pass (typically few Hz) while reducing high frequency noise. Neglecting the effect of input current and voltage offset in the TIA, the measured current through the working electrode *I* is obtained from the measured output voltage:

(18) $$I=\frac{\left(V_{out}-V_{REF}\right)}{R_{f}}$$

where $V_{REF}$ is the voltage at the positive input of the TIA which is equal to the WE potential since the OpAmp works in linear mode. The output voltage is discretized with a 10-bit analog-to-digital converter. The total current consumption of the tag is approximately 1300 μA. The potentiostat allows to perform various amperometric techniques, such as cronoamperometry and cyclic voltammetry. In the latter, the variable bias between the electrodes is obtained programming the DAC output $V_{DAC}$:

(19) $$V_{BIAS}=V_{WE}-VRE=\frac{R_{3}}{R_{3}+R_{4}}V_{DAC}-\frac{R_{1}}{R_{1}+R_{2}}V_{EN}$$

where $V_{EN}$ is the voltage at a digital output of the microntroller that is used to activate the bias in the pontentiostat. Cyclic voltammetry is a powerful characterization technique able to provide valuable information of an electrochemical system, such as for example the potential required to either oxidize or reduce a target electroactive species. Cyclic voltammetry requires sweeping the applied potential what reduces its interest for PoCT devices and is mainly used to characterize the sensor in various sensor development stages.

## 4. Glucose Sensor

**Figure 14:**
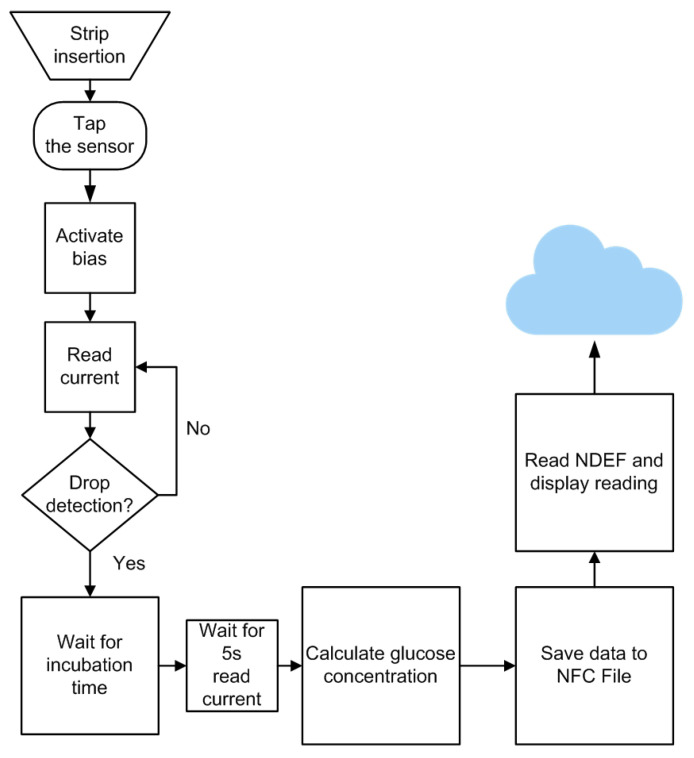
Firmware flowchart.

**Figure 15:**
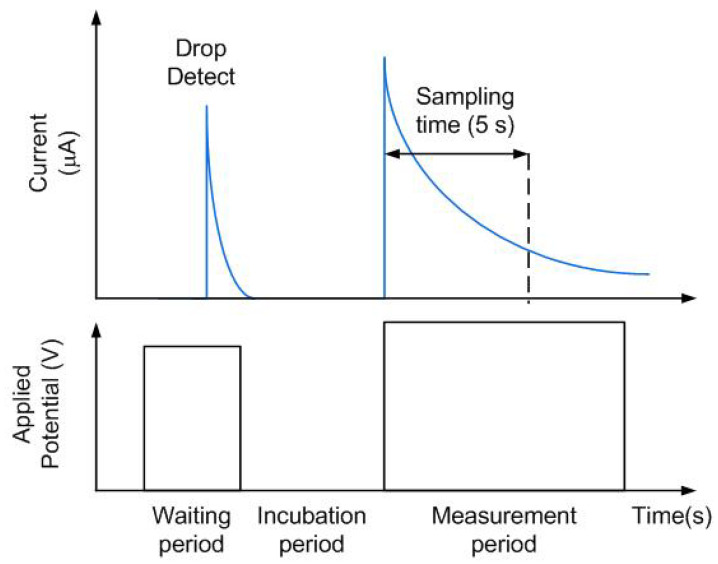
Test strip timing sequence.

In this work, commercial low-cost glucose test strips from Sinocare are used as the electrodic system, including an enzymatic sensor as working electrode. The enzyme glucose oxidase catalyzes the chemical reaction of glucose in the presence of oxygen, increasing the pH, decreasing the partial pressure of oxygen, and producing hydrogen peroxide and gluconic acid. The test strips are designed to work at an applied bias of 0.4 V, set by the redox mediator used to reduce the required oxidation potential, and with small sample volumes (typically 0.6 μL). The test strips draw the solution placed in one of their ends by capillarity. The reaction begins when the drop reaches the active enzymatic region, and stabilizes after 5 s. The amperometric measurement at that moment corresponds to the final reading that has to be compared with the values in a look-up table to obtain the glucose concentration in mg/dL [44,45]. The applied potential is key to determine the sensitivity and selectivity of the biosensor and therefore must be carefully selected. A glucose stock solution (0.2 M) was prepared with distilled water and kept at room temperature for 24 h prior to use to ensure the presence of β-D-glucose form. After that, various concentrations within the measurement range were prepared by dissolving of the stock solution in distilled water to be analyzed by the sensor. The steps shown in the flowchart of Figure 14 have been implemented in the firmware of the microcrontroller according to the time sequence of the test strip schematically described in Figure 15. The main steps are:

1. Insert strip into the potentiostat without the sample.
2. Approach the smartphone (NFC enabled) close to the NFC sensor. When the smartphone is placed over the sensor the electronics is turned on.
3. The microcontroller biases the potentiostat and measures the current, waiting for a change that indicates the presence of a drop.
4. The user introduces the sample by depositing a drop on the opening of the strip.
5. The sample fills the chamber by capillary action.
6. When the presence of the drop is detected, the microcontroller turns off the cell, and starts the incubation time (52 ms). The incubation time allows the chemicals to dissolve, the enzymatic reaction to occur and the solution to become homogeneous.
7. When incubation time is completed, the potential required to electrochemically oxidize the mediator that chemically reacts with the active site of the enzyme, bringing it back to its original oxidation state, is applied The current is monitored and its final value is interpolated in a previously obtained calibration curve. Then the concentration value is reported, saving the data in the NFC IC EPPROM via I2C.
8. The smartphone reads the concentration value stored in the memory and displays the result in the user’s screen, uploading the measurement into a database for later consultation.

## 5. Results

**Figure 16:**
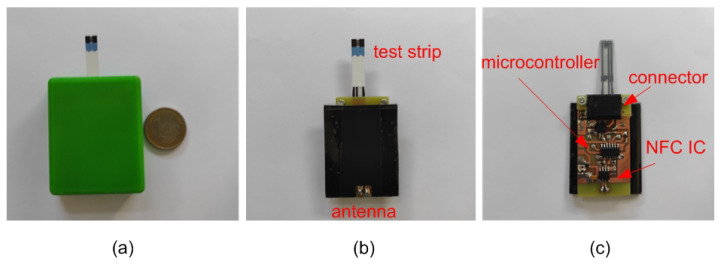
NFC glucometer prototype. (**a**) Prototype including protection case, (**b**) top view, and (**c**) bottom view.

**Figure 17:**
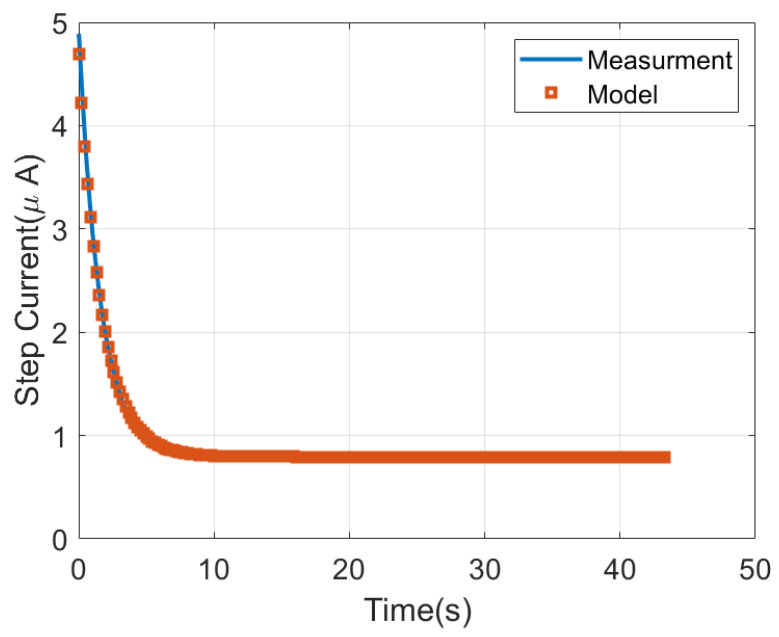
Measured current as a function of time for the dummy cell when a step voltage is applied.

This section presents the results measured with the proposed platform. To this end a prototype has been manufactured. Figure 16 shows a photograph of the prototype designed. A polylactic acid (PLA) 3D casing was printed to protect the electronics, including a custom connector for the test strip. The cost of the prototype is about EUR 4. In a first step, measurements with a dummy cell have been performed, in order to check the accuracy of the potentiostat. The dummy cell is based on a passive electronic network that corresponds to the Randles equivalent circuit of Figure 2, with $R_{s}$ = 200 kΩ, $R_{p}$ = 1000 kΩ and $C_{p}$ = 10 μF. As the current is controlled by the step voltage and the impedance between the working and reference electrode, a simplified equivalent circuit can be considered for the branch between the reference and the counter electrodes ($Z_{RC}$) to reduce the number of components. In the dummy cell, $Z_{RC}$ is modelled with a resistor of value $R_{RC}$ = 100 kΩ. Typically, the nominal tolerance of the components is 10%. A step voltage is applied and the current is measured with the potentiostat. For each point 16 ADC measurements have been averaged in order to reduce noise. Figure 17 compares the step response obtained in the measurements to the theoretical model given by (2) for a voltage step of 1 V and the gain set to 240 kΩ. Good agreement is found verifying the accuracy of the measurements within the range of typical currents of electrochemical sensors.

Once the system is verified with the dummy device, glucose concentration measurements are performed with the NFC potentiostat used as battery-less glucometer with the commercial test strips from Sinocare. The parameters of the potentiostat have been adjusted for this specific sensor. The gain of the potentiostat is set to 100 kΩ to avoid the saturation of the amplifier, and the bias is set to 0.4 V adjusting the DAC output. The resistance used in the voltage dividers of the potentiostat (Figure 13) were $R_{1}$ = 22 kΩ, and $R_{2}$ = $R_{3}$ = $R_{4}$ = 100 kΩ. Glucose concentration (C) is determined from the measured current (I) and the calibration data using a regression equation:

(20) C(mg/dL)=37.68·I(μA)−22.30

**Figure 18:**
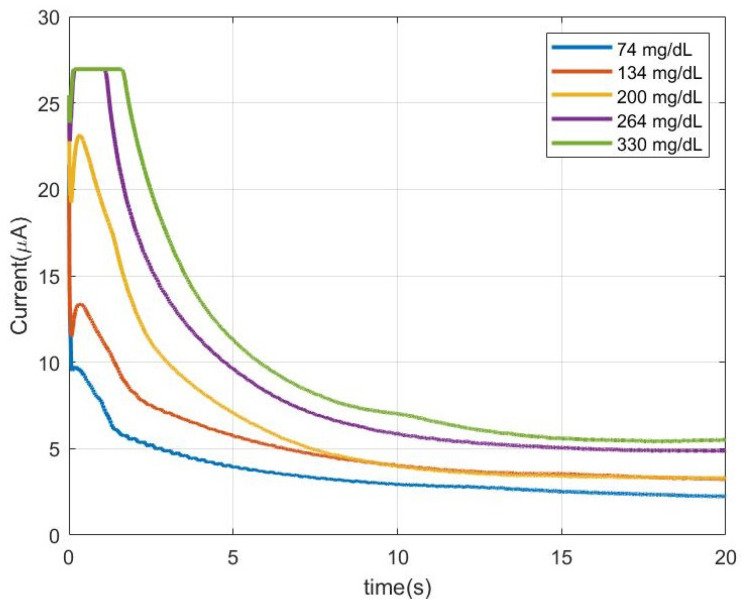
Measured current as a function of time for different glucose concentrations.

Calibration data was obtained by measuring solutions with different concentrations of glucose between 60 mg/dL and 425 mg/dL. Typical time response is shown in Figure 18. These measurements demonstrate the clear dependence between current and glucose concentration. The test strips have been designed in such a way that after 5 s the sample reaches the active zone of the sensor, and the enzymatic reaction is at its maximal reaction rate. Therefore, the measurement of the concentration is performed at that precise moment. Note that for very high concentrations, amplifier saturation could occur during the first part of the measurement. However, this phenomenon has no impact since the value of interest is the one measured after 5 s, in which the amplifier is not saturated. To avoid saturation, the gain could be decreased at the cost of losing sensitivity when testing low concentrations. A program in the microcontroller that implements the flowchart described in the last section has been developed.

**Figure 19:**
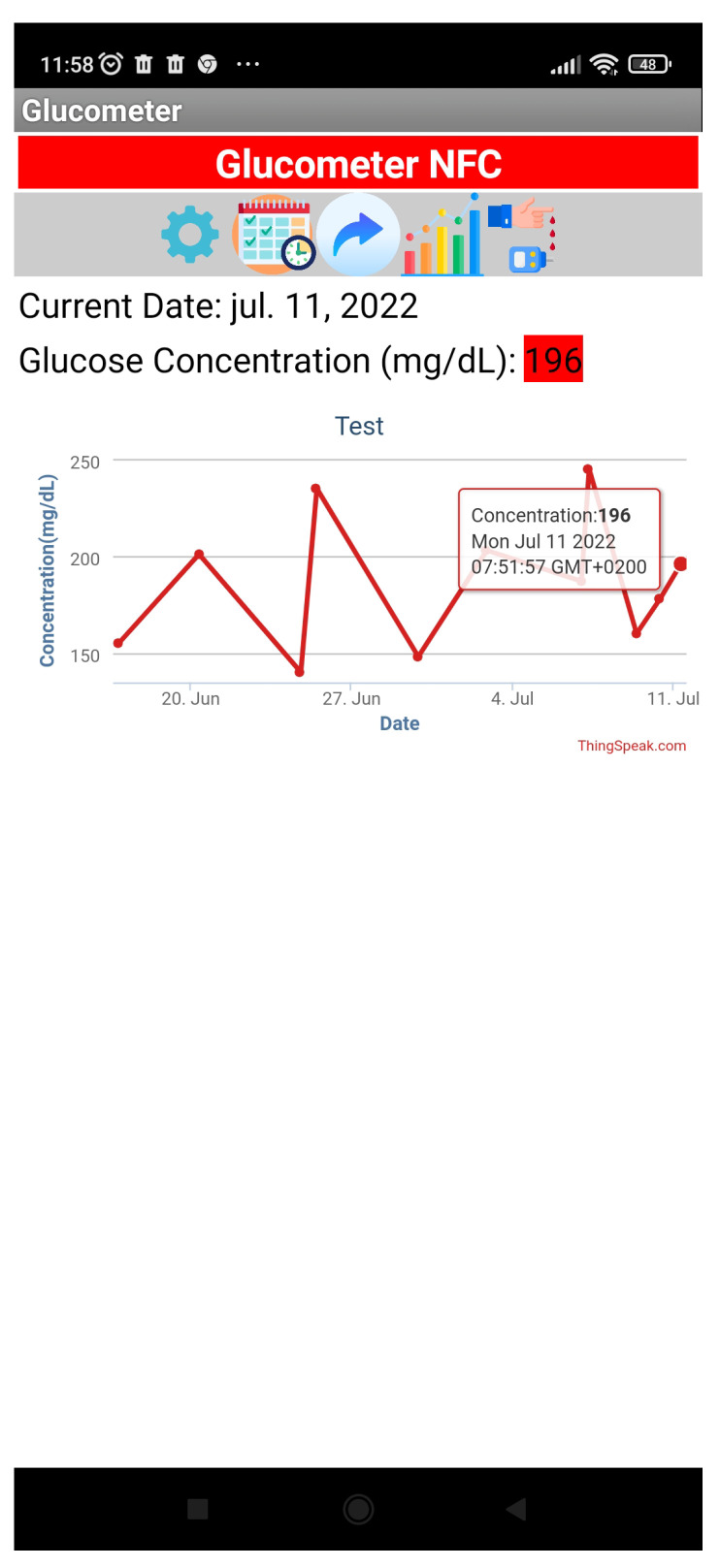
Smartphomneapp screen used to record glucometer data.

An application under the Android operating system has been developed to read the NFC tag, including some tools such as the ability to upload the measured data to a database (see Figure 19). Here the Thingspeak platform from Mathworks is used to save the historical data using REST API protocol. The application provides voice and text messages to guide the user, including calendar reminders. Note that the reading is stored in standard text NFC Data Exchange Format (NDEF), therefore it can be read using standard NFC applications.

**Figure 20:**
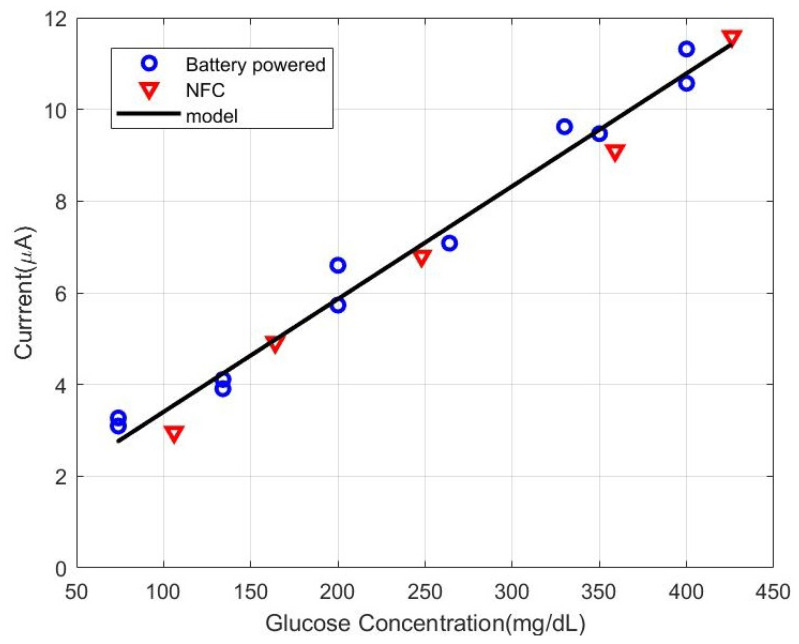
Measured current after 5 s as a function of glucose concentration.

A sample of the output current is taken for each solution at 5 s and compared with the reading obtained by the Sinocare commercial glucometer. Linear regression is performed to fit the data. The results are shown in Figure 20. The firmware contains the linear equation that allows calculating the glucose concentration from the current obtained from the average reading of the ADC at the potentiostat output using (18). Measurements were made both with the potentiostat externally powered and through the harvesting of energy from a mobile, obtaining results with a high degree of agreement. It must be considered that since the test strip is for a single use, different test strips are used for each measured concentration. A correlation coefficient (R${}^{2}$) of 99% was found between the commercial glucometer and the proposed potentiostat. Since a blood sugar level below 140 mg/dL (7.8 mmol/L) is considered normal and a level above 200 mg/dL (11.1 mmol/L) taken two hours after a meal is an indicator of diabetes [46], the proposed prototype covers the range of interest.

## 6. Conclusions and Future Work

Most current smartphones include an NFC reader, mainly for making payments, but they can also be used as readers for several sensor applications. To this end, low-cost NFC IC with energy harvesting capabilities from different NFC manufacturers are available on the market. The combination of these NFC ICs and low-power microcontrollers allows the development of green Internet of Things (IoT) applications based on battery-less NFC sensors. The standardization of data interface exchange based on NDEF format allows the development of simple but powerful mobile-based applications. In this work, the design of a battery-less NFC potentiostat for amperometric measurements using electrochemical sensors has been presented. It consists of measuring the current at the output of a high-gain transimpedance amplifier when a step bias is applied to the potentiostat. Due to its simplicity, it is a very good candidate method to be used in PoCT devices. The concentration of a target analyte is measured at a specific time and compared to a previously obtained calibration curve. Since electrochemical sensors have slow dynamics, long time intervals (typically several seconds) are needed to perform amperometric measurements. Measuring low currents through electrochemical sensors requires a stable power supply; this is a challenge when integrated in NFC-powered devices which has been addressed in this work. To perform the measurements, the user usually places the mobile on top of the tag, therefore the tag must be designed to be robust against variations in the inductance value caused by the metal parts of the mobile. To ensure stable bias, the solution proposed is an antenna loop covered with ferrite sheets. As a proof of concept, a prototype has been integrated into a housing to measure glucose concentration using commercial test strips. The measurements obtained are compared to those provided by a commercial glucometer, showing excellent correlation. On the one hand, the number of components of the prototype is reduced as no battery is needed. On the other hand, the reading eludes the use of screens. As a result, the cost of future PoCT devices is reduced, and the ease of use improved, the latter being an important requirement for both the elderly and future remote monitoring applications such as non-invasive glucose level measurement from interstitial fluid [47]. Potentiostat parameters can be configured to measure voltages up to 1 V and currents below μA depending on the sensor. The design can be extended to other applications harnessing a broad range of electrochemical sensors, including flexible ones.

## Figures and Tables

**Table 1:** Measured antenna parameters at 13.56 MHz at different conditions.

Parameter	Free Space	at 0.5 mm Metal	with Ferrite Sheet and Metal
Number of turns N	5	5	5
Dimmensions	30 mm × 30 mm	30 mm × 30 mm	30 mm × 30 mm
Width	0.5 mm	0.5 mm	0.5 mm
Gap	0.5 mm	0.5 mm	0.5 mm
Inductance $L_{a}$	1.26 μH	0.55 μH	2.47 μH
Quality factor $Q_{a}$	90.7	33.6	35.7
Resonance frequency $f_{r}$	85 MHz	121 MHz	48 MHz

## Data Availability

The data presented in this study are available on request from the corresponding author.
